# Early-mid gestation as a critical predictive window for infantile atopic dermatitis: A longitudinal multi-matrix metabolomic study^☆^

**DOI:** 10.1016/j.waojou.2026.101452

**Published:** 2026-08-26

**Authors:** Xi Fu, Zekai Liang, Chuchu Luo, Yuwei Tang, Xin Zhang, Guo-Qing Fang, Da-Yong Zha, Yu Sun

**Affiliations:** a Guangdong-Hong Kong-Macao Joint Laboratory for Contaminants Exposure and Health, School of Public Health, Guangdong Pharmaceutical University, Guangzhou, 510006, PR China; b State Key Laboratory of Swine and Poultry Breeding Industry, Guangdong Provincial Key Laboratory for the Development Biology and Environmental Adaptation of Agricultural Organisms, College of Life Sciences, South China Agricultural University, Guangzhou 510642, Guangdong, PR China; c Baiyun Branch of the Third Affiliated Hospital of Guangzhou Medical University, Guangzhou Baiyun Maternal and Child Health Hospital, Guangzhou, Guangdong 510400, PR China

**Keywords:** Atopic dermatitis, Metabolomics, Prenatal prediction, Machine learning, Early gestation

## Abstract

**Introduction:**

Atopic dermatitis (AD) is a prevalent inflammatory skin disease with origins increasingly traced to the intrauterine environment. While maternal diet and immunity influence infant health, the optimal time window and specific metabolic signatures conferring protection against AD remain unclear. This study aims to characterize longitudinal perinatal metabolic profiles to identify stage-specific predictive biomarkers and critical windows for fetal metabolic exposure.

**Methods:**

A longitudinal birth cohort study was conducted with 109 mother-infant pairs recruited between 2021 and 2023. Biological samples including maternal plasma (collected at early-mid gestation and pre-delivery), umbilical cord blood, and meconium were analyzed using untargeted LC-MS metabolomics. Infants were followed for 1 year to ascertain AD status (AD group n = 39; Healthy group n = 70). Logistic regression was performed to identify significant metabolites associated with health outcomes, and Random Forest classification was utilized to compare predictive performance across different developmental windows.

**Results:**

Distinct metabolic phenotypes were identified. In early-mid gestation maternal plasma, the Healthy group exhibited a significant enrichment of protective metabolites across 2 major classes: Polyunsaturated Fatty Acids (PUFAs), including 13S-HODE (OR = 0.11, P < 0.001) and Alpha-Linolenic Acid (OR = 0.51, P = 0.014); and Flavonoids, including Formononetin (OR = 0.33, P = 0.005), Equol (OR = 0.48, P = 0.012), and Quercetin (OR = 0.87, P = 0.027). Fewer metabolic alterations were observed in subsequent phases, such as ergocalciferol (OR = 0.56, P = 0.028) in late gestation and isovitexin (OR = 0.88, P = 0.037) in meconium. In cord blood, key predictive markers included phenyl acetate (OR = 0.28, P = 0.025), the antioxidant Ribose-1,5-bisphosphate (OR = 0.09, P = 0.017), and sterculic acid (OR = 1.23, P = 0.026). Notably, the latter 2 were consistently identified across both early-mid gestation maternal plasma and cord blood. Crucially, Random Forest modeling revealed that integrating early-mid gestation metabolites significantly improved AD prediction over the Baseline Clinical Model (AUC 0.831, 95% CI 0.73–0.93 vs. AUC 0.463, 95% CI 0.35–0.57), vastly outperforming late gestation metabolites (AUC 0.669, 95% CI 0.48–0.86). In neonates, the cord blood model (AUC 0.817, 95% CI 0.69–0.95) demonstrated superior predictive value compared to meconium (AUC 0.644, 95% CI 0.46–0.83). This predictive pattern remained highly robust in parsimonious models restricted to only 6 core metabolites.

**Conclusion:**

This study highlights potential protective and risk perinatal metabolic profiles, suggesting that the early maternal metabolic milieu plays a critical role in ‘priming’ the infant immune system against AD and identifying early-mid gestation as an optimal window for early risk prediction. These predictive associations require confirmation in future mechanistic and interventional trials before informing targeted nutritional strategies.

## Introduction

Atopic dermatitis (AD) is a prevalent chronic inflammatory skin disorder characterized by intense pruritus, xerosis, and eczematous lesions.^1^^,^^2^ Beyond the immediate clinical burden, infantile AD imposes considerable emotional and economic stress on families and often serves as the initial phase of the “atopic march,” progressing to asthma and allergic rhinitis.^3^^,^^4^ Notably, the global incidence of early-onset infantile AD is increasing, with approximately 60% of cases emerging within the first year of life, and 45% before 6 months of age.^5^^,^^6^ This striking trend of early-life onset strongly suggests that susceptibility to AD is heavily programmed before birth, pointing toward the intrauterine environment and perinatal factors as critical determinants of infant skin and immune health.

While AD pathogenesis involves a complex interplay of genetic predisposition, immune dysregulation, and environmental exposures,^7^, ^8^, ^9^, ^10^, ^11^, ^12^ emerging evidence highlights the profound impact of the metabolic environment on immune development. Metabolomics offers a high-resolution, comprehensive framework to detect low-molecular-weight molecules and understand their functional roles in biological and disease mechanisms.^13^^,^^14^ Previous studies have utilized metabolomic approaches to explore these associations. For instance, Ilves et al analyzed metabolic disparities in the skin and blood of patients with,^15^ and Du et al examined metabolites in maternal blood and feces related to the development of infantile.^16^

Despite these advancements, significant knowledge gaps remain in understanding the perinatal metabolic origins of AD. First, it is entirely unclear which developmental stage—such as early pregnancy, late pregnancy, or the immediate neonatal period—represents the most critical and predictive time window for AD programming. Second, the specific metabolites or structural categories (eg, dietary lipids, flavonoids) that act as key drivers of risk or confer protection remain poorly defined. Third, current research often isolates either maternal or infant samples, lacking a comprehensive, longitudinal multi-matrix approach. Consequently, the dynamics of maternal-fetal metabolic programming and how the early maternal systemic milieu shapes the neonatal metabolome are largely unknown.

To address these critical gaps, we conducted a longitudinal birth cohort study utilizing untargeted LC-MS metabolomics across 4 distinct perinatal biological matrices: maternal plasma during early-mid gestation, maternal plasma in late gestation, umbilical cord blood, and neonatal meconium. By systematically profiling these matrices, we aimed to Ref. 1 identify stage-specific key protective and risk metabolic group biomarkers,^2^ determine the optimal predictive time window for infantile AD through machine learning. This study provides crucial objective evidence for early risk identification and establishes a scientific foundation for precision nutritional interventions during pregnancy to “prime” the infant immune system against AD.

## Materials and methods

### Study design

**Fig. 1:**
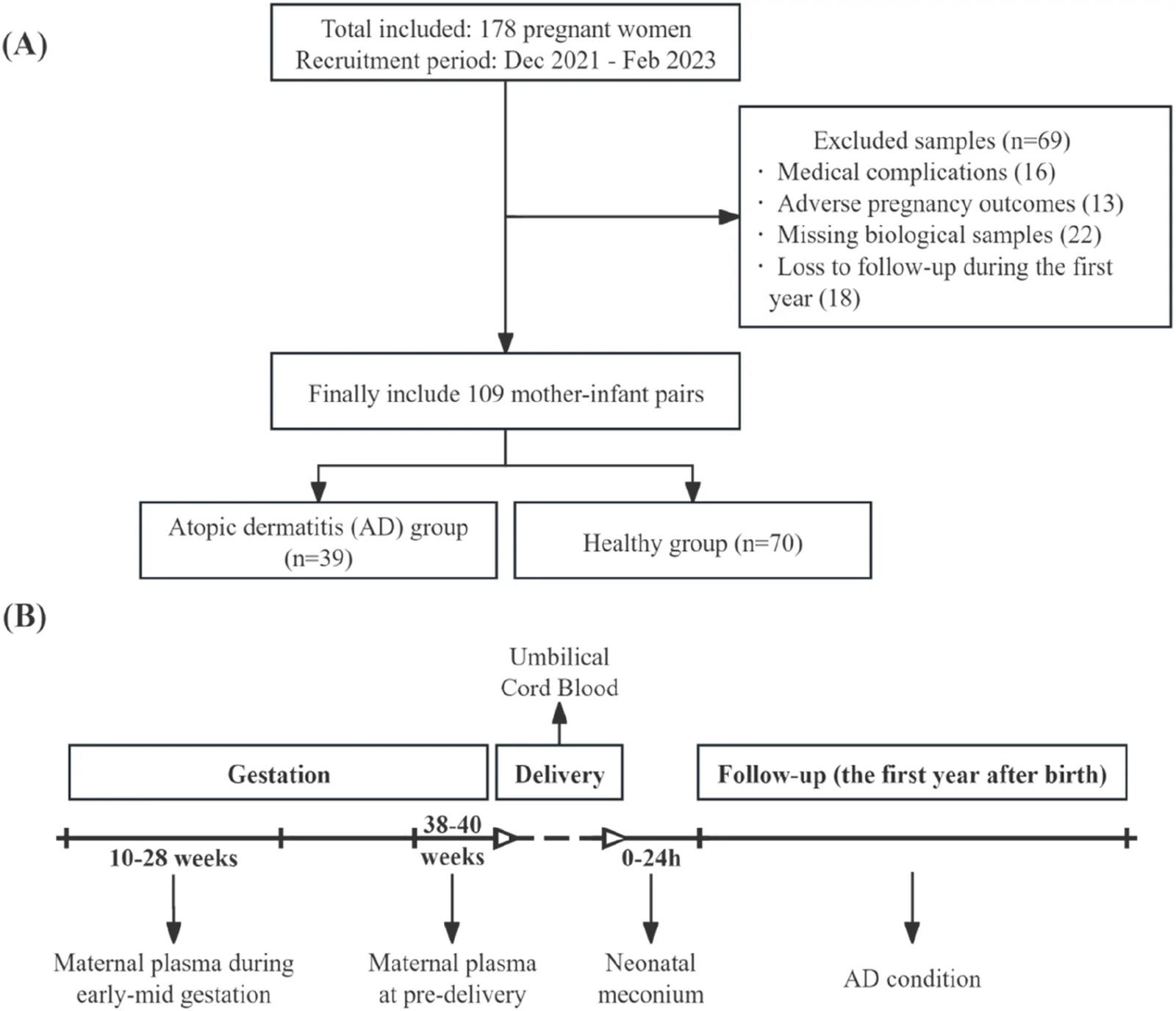
**Overview of the longitudinal birth cohort study design.** (A) Participant flow diagram detailing the screening, exclusions, and final inclusion of 109 mother-infant pairs, prospectively followed for 1 year and classified into the Atopic Dermatitis (AD) group (n = 39) and Healthy group (n = 70). (B) Timeline of biological sample collection across 4 distinct perinatal matrices: maternal plasma during early-mid gestation (10–28 weeks) and late gestation (38–40 weeks), followed by umbilical cord blood and neonatal meconium collected at birth

This longitudinal birth cohort study involved 109 pregnant women recruited in Guangdong between December 2021 and February 2023. Infants received follow-up assessments at least once every 3 months throughout their first year of life to collect data on neonatal characteristics and health outcomes. The diagnosis of AD in infants during the one-year follow-up was conducted by pediatricians with specialized training in pediatric dermatology, strictly applying the Williams criteria for atopic dermatitis, which include evidence of an itchy skin condition plus major criteria such as typical morphology and distribution, and a history of skin flexure involvement. Infant participants were subsequently classified into the AD group (n = 39) or the Healthy group (n = 70; Fig. 1).

### Inclusion and exclusion criteria

The inclusion criteria for pregnant women were: (1) maternal age between 18 and 35 years; (2) enrollment during early-mid gestation (between 10 and 28 gestational weeks); and (3) provision of informed consent to participate in the study. Exclusion criteria included: (1) neonates with birth asphyxia (Apgar score <8); (2) infants suffering from congenital malformations or diseases that impact the immune system (eg, congenital heart disease or inherited metabolic disorders); (3) pregnant women with autoimmune disorders (eg, systemic lupus erythematosus, rheumatoid arthritis, Graves' disease); and (4) participants with missing follow-up data or incomplete biological samples. Furthermore, all pregnant women maintained a standard, unrestricted daily diet; mothers following specific dietary restrictions (such as avoiding dairy or eggs for allergy prevention) were excluded to observe the natural metabolic profile."

### Collection of biological samples

The biological samples collected for this study consisted of maternal plasma, umbilical cord blood, and meconium. Maternal plasma was sampled at 2 distinct stages: early-mid gestation (between the 10th and 28th weeks) and late gestation (between the 38th and 40th weeks, immediately prior to delivery). Umbilical cord blood was acquired immediately following delivery, and meconium was collected within 24 h of birth. All biological specimens were preserved in freezers at −80 °C.

### Metabolomic profiling of biological samples

We evaluated the chemical compounds in maternal plasma, umbilical cord blood, and meconium through untargeted liquid chromatography-mass spectrometry (LC-MS). For sample preparation, 50 mg of meconium or 50 μL of plasma was mixed with 600 μL of a methanol solution containing 2-Amino-3-(2-chloro-phenyl)-propionic acid (4 ppm). The mixture was centrifuged at 12,000 rpm for 10 min at 4 °C, and 300 μL of the resulting supernatant was filtered through a 0.22 μm membrane. Liquid chromatography analysis was conducted using a Vanquish UHPLC System (Thermo Fisher Scientific, USA). Chromatographic separation utilized an ACQUITY UPLC® HSS T3 column (150 × 2.1 mm, 1.8 μm; Waters, Milford, MA, USA) maintained at 40 °C, with a flow rate of 0.3 mL/min and an injection volume of 2 μL. Mass spectrometric detection of metabolites was performed on an Orbitrap Exploris 120 (Thermo Fisher Scientific, USA) using an electrospray ionization (ESI) source. Data were acquired in both positive and negative ion modes, with spray voltages of 3.50 kV and −2.50 kV, respectively. The sheath gas was set to 40 arb, the auxiliary gas to 10 arb, and the capillary temperature to 325 °C. The mass spectrometer operated at a resolution of 60,000 for primary mass spectrometry (MS1) with a detection range of m/z 100 to 1000, while the resolution for secondary mass spectrometry (MS2) was 15,000. Finally, a normalized collision energy of 30% was employed, and dynamic exclusion was applied to eliminate redundant MS/MS data.

To monitor instrument stability and data reliability, pooled Quality Control (QC) samples—created by combining equal aliquots from all biological samples within a matrix—were injected at the beginning of the run and after every 10 experimental samples. Raw data processing included missing-value handling, where features with >20% missing values in biological samples were removed, and remaining missing values were imputed using the K-Nearest Neighbors (KNN) method. Data were then subjected to median normalization, log10 transformation, and Pareto scaling. Finally, to ensure analytical robustness, unstable features exhibiting a Relative Standard Deviation (RSD) > 30% across the QC samples were strictly filtered out prior to statistical analysis.

The molecular weight of metabolites was determined from the mass-to-charge ratio (m/z) of precursor ions in MS1 spectra. Molecular formula prediction was conducted based on mass accuracy (ppm) and adduct ion information, followed by matching with the Human Metabolome Database (HMDB)^17^ for level 1 identification. For metabolites with MS2 spectra acquired in the quantitative list, their fragment ions were matched against the corresponding entries in the database for level 2 identification. Differential metabolites were then subjected to functional pathway enrichment analysis using the R package MetaboAnalyst.^18^

### Statistical analyses

All statistical analyses were performed using R software. Demographic and clinical characteristics were compared between the AD group and the healthy group using the chi-square test or Fisher's exact test. Differential metabolites were screened using a rigorous multi-step selection strategy. First, only metabolites with confident structural annotation (Level 2 identification via MS/MS spectral matching) were retained. Second, univariate filtering was applied to identify significantly altered compounds (Mann–Whitney *U* test, P < 0.05, VIP>1, Fold Change >1.2 or <0.83). Third, binary logistic regression models (adjusted for maternal age, BMI, and parental allergy) were utilized to determine the association between candidate metabolites and AD risk. P-values in binary logistic regression models were adjusted using the false discovery rate (FDR) method, with an FDR-adjusted P < 0.05 considered statistically significant. These specific confounders (maternal age, BMI, and parental allergy) were selected because they represent established, universally documented baseline risk factors that are definitively known to clinicians during the early prenatal window, ensuring our predictive models remain clinically applicable for early-gestation screening. To evaluate stage-specific maternal-fetal metabolic correlations, Spearman correlation coefficients (R) and P-values were calculated based on quantitative measurements across the distinct biological matrices.

Random Forest classification was conducted using the randomForest package in R to evaluate the predictive potential of metabolites at distinct developmental windows. First, a Baseline Clinical Model was constructed using baseline demographic and familial factors (maternal age, pre-pregnancy BMI, and parental allergy) as predictors. Subsequently, 4 matrix-specific comprehensive models (Early-Mid Gestation, Late Gestation, Umbilical Cord Blood, and Meconium) were built by incorporating all significant differential metabolites from their respective matrices alongside the conventional risk factors. To eliminate statistical bias caused by the unequal number of candidate biomarkers across different matrices, parsimonious models were further constructed by restricting the metabolic features to a maximum of the top 6 most contributing metabolites (ranked by Variable Importance scores). To maximize statistical power and mitigate overfitting in our moderately sized cohort (N = 109), models were evaluated using Leave-One-Out Cross-Validation (LOOCV). To further ensure model stability against high-dimensional omics data, a five-fold cross-validation repeated 10 times was conducted as a sensitivity analysis. 95% Confidence Intervals (CIs) for the Area Under the Curve (AUC) were calculated using the DeLong method. Comparisons of AUC between models were performed using the bootstrap test via the pROC package, with P < 0.05 indicating a statistically significant difference.

## Result

### Demographic and clinical characteristics

A total of 178 pregnant women were recruited between December 2021 and February 2023. Participants were excluded due to adverse pregnancy outcomes (n = 13), medical complications (n = 16), missing biological samples (n = 22), or loss to follow-up during the first year (n = 18). The final analysis included 109 mother-infant pairs with complete biological samples and AD outcomes, classified into the AD group (n = 39) and the Healthy group (n = 70). Biological samples, including maternal plasma (early-mid and late gestation), umbilical cord blood, and neonatal meconium, were collected at different stages (Fig. 1). The incidence rate of AD in the first year of life was 35.8%. Demographic and clinical characteristics exhibited no significant differences between the AD group and the healthy group (p > 0.05; Table 1). Notably, traditional risk factors such as parental allergy and mode of delivery were comparable between the 2 groups, underscoring the limitations of relying solely on baseline clinical characteristics for early AD prediction and highlighting the need for molecular biomarkers.

**Table 1:** Demographic and Clinical Characteristics of the Study Population.

Characteristics	Total (N = 109)	AD group (n = 39)	Healthy group (n = 70)	*P*-value
**Maternal age (years)**	30.1 ± 4.2	30.0 ± 4.1	30.2 ± 4.2	0.613
<35, n (%)	91 (83.5)	34 (87.2)	57 (81.4)	
≥35, n (%)	18 (16.5)	5 (12.8)	13 (18.6)	
**Pre-pregnancy BMI (kg/m ^2^)**	21.8 ± 4.3	21.8 ± 5.1	21.8 ± 3.8	0.164
Underweight (<18.5), n (%)	21 (19.3)	11 (28.2)	10 (14.3)	
Normal (18.5–23.9), n (%)	70 (64.2)	21 (53.8)	49 (70.0)	
Overweight/Obese (≥24), n (%)	18 (16.5)	7 (18.0)	11 (15.7)	
**Gestational age at sampling (weeks)**	17.2 ± 6.5	18.3 ± 6.1	16.7 ± 6.7	0.325^a^
**Education level**				0.907
Middle school or below	32 (29.4)	11 (28.2)	21 (30.0)	
High school/Technical	20 (18.3)	8 (20.5)	12 (17.1)	
College/University	57 (52.3)	20 (51.3)	37 (52.9)	
**Household income (RMB/month)**				0.818
≤5000	29 (26.6)	9 (23.1)	20 (28.6)	
5001–10,000	30 (27.5)	11 (28.2)	19 (27.1)	
>10,000	50 (45.9)	19 (48.7)	31 (44.3)	
**Mode of delivery**				0.692
Vaginal	80 (73.4)	30 (76.9)	50 (71.4)	
Cesarean section	29 (26.6)	9 (23.1)	20 (28.6)	
**Parental allergy**				0.569
Yes	18 (16.5)	8 (20.5)	10 (14.3)	
No	91 (83.5)	31 (79.5)	60 (85.7)	
**Lifestyle factors**				
Smoking during pregnancy, n (%)	4 (3.7)	0 (0.0)	4 (5.7)	0.295
Alcohol consumption, n (%)	2 (1.8)	0 (0.0)	2 (2.9)	0.536
Passive smoking exposure, n (%)	39 (35.8)	12 (30.8)	27 (38.6)	0.544
**Infant characteristics**				
**Infant gender (male)**	60 (55.0)	24 (61.5)	36 (51.4)	0.414
**Birth weight (kg)**				0.752
Mean ± SD	3.1 ± 0.4	3.2 ± 0.4	3.1 ± 0.4	
≤2.5 or ≥4.0, n (%)	12 (11.0)	5 (12.8)	7 (10.0)	
Normal (2.5–4.0), n (%)	97 (89.0)	34 (87.2)	63 (90.0)	

Data are presented as n (%) for categorical variables and Mean ± SD for continuous variables. P-values for categorical variables were calculated using the Fisher's exact test. P-values for continuous variables were calculated using the Mann-Whitney *U* test

a Using T-test to calculate the P-value of Gestational Age at Sampling (weeks).

### Metabolic profiling and identification of characteristic metabolites

Following initial LC-MS data acquisition and rigorous quality control filtering, the data attrition funnel—from total raw features detected to the final confidently annotated metabolites included in the statistical analysis—is summarized for each biological matrix (Supplementary Table 1). Notably, maternal plasma and cord blood matrices were processed within a unified analytical batch, yielding 547 confidently annotated metabolites, while the independent meconium matrix yielded 746 annotated metabolites.

**Fig. 2:**
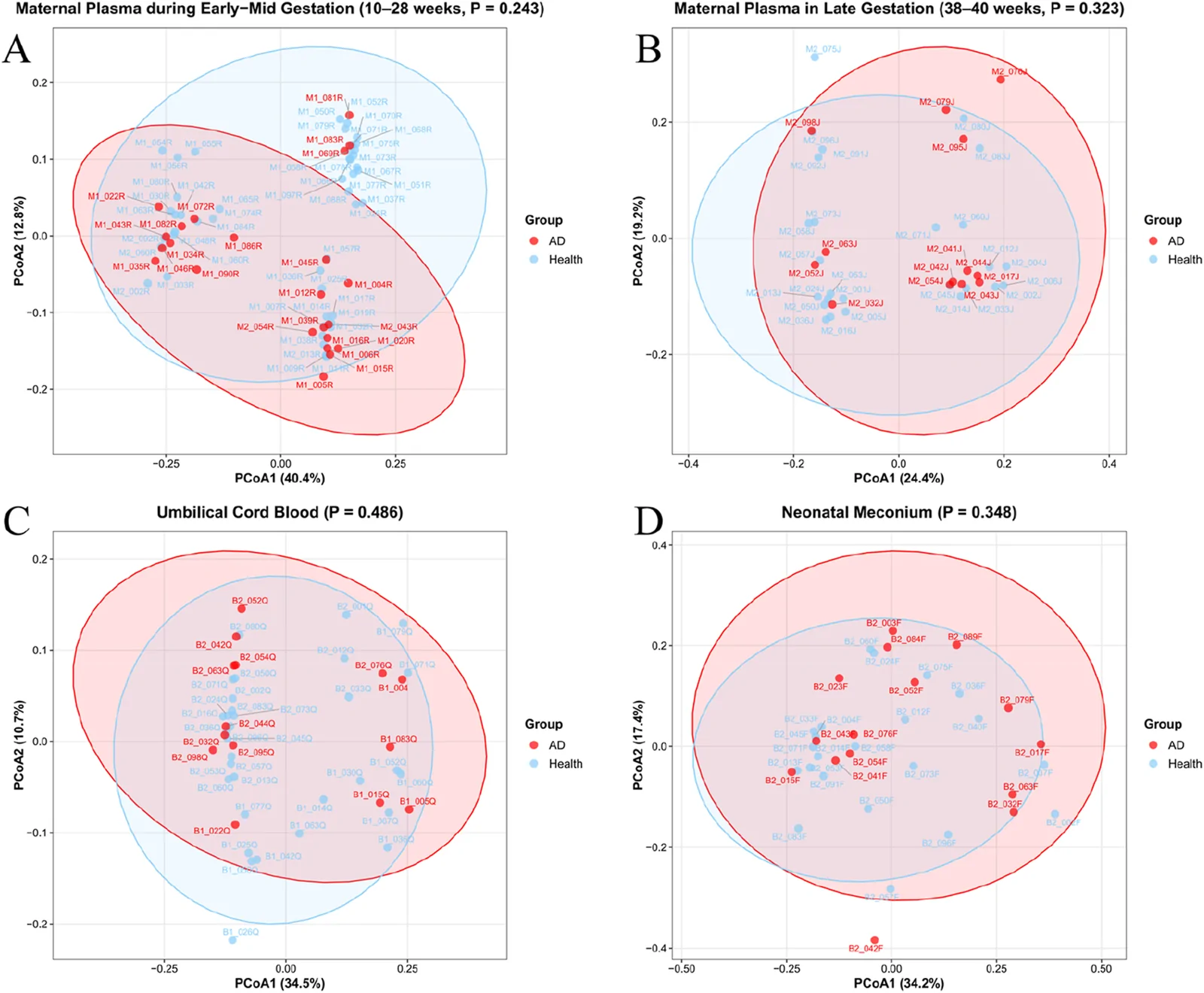
**Principal Coordinate Analysis (PCoA) diagrams of the global metabolome.** Unsupervised PCoA was performed using the relative abundance of all annotated metabolites to visualize the global metabolic composition between the Atopic Dermatitis and Healthy groups across 4 matrices: (A) Maternal Plasma during Early-Mid Gestation (10–28 weeks); (B) Maternal Plasma in Late Gestation (38–40 weeks); (C) Umbilical Cord Blood; and (D) Neonatal Meconium. Each point represents an individual sample. Ellipses represent the 95% confidence intervals for each group

As shown in Fig. 2, unsupervised Principal Coordinate Analysis (PCoA) incorporating all annotated metabolites revealed that the global metabolic composition largely overlapped between the AD and Healthy groups across all 4 perinatal stages (PERMANOVA P > 0.05). This overlap indicates that general maternal-fetal metabolic homeostasis is fundamentally maintained. However, despite this global stability, our subsequent rigorous multi-step differential analysis identified highly specific, localized metabolic shifts that diverge significantly between the 2 groups.

**Fig. 3:**
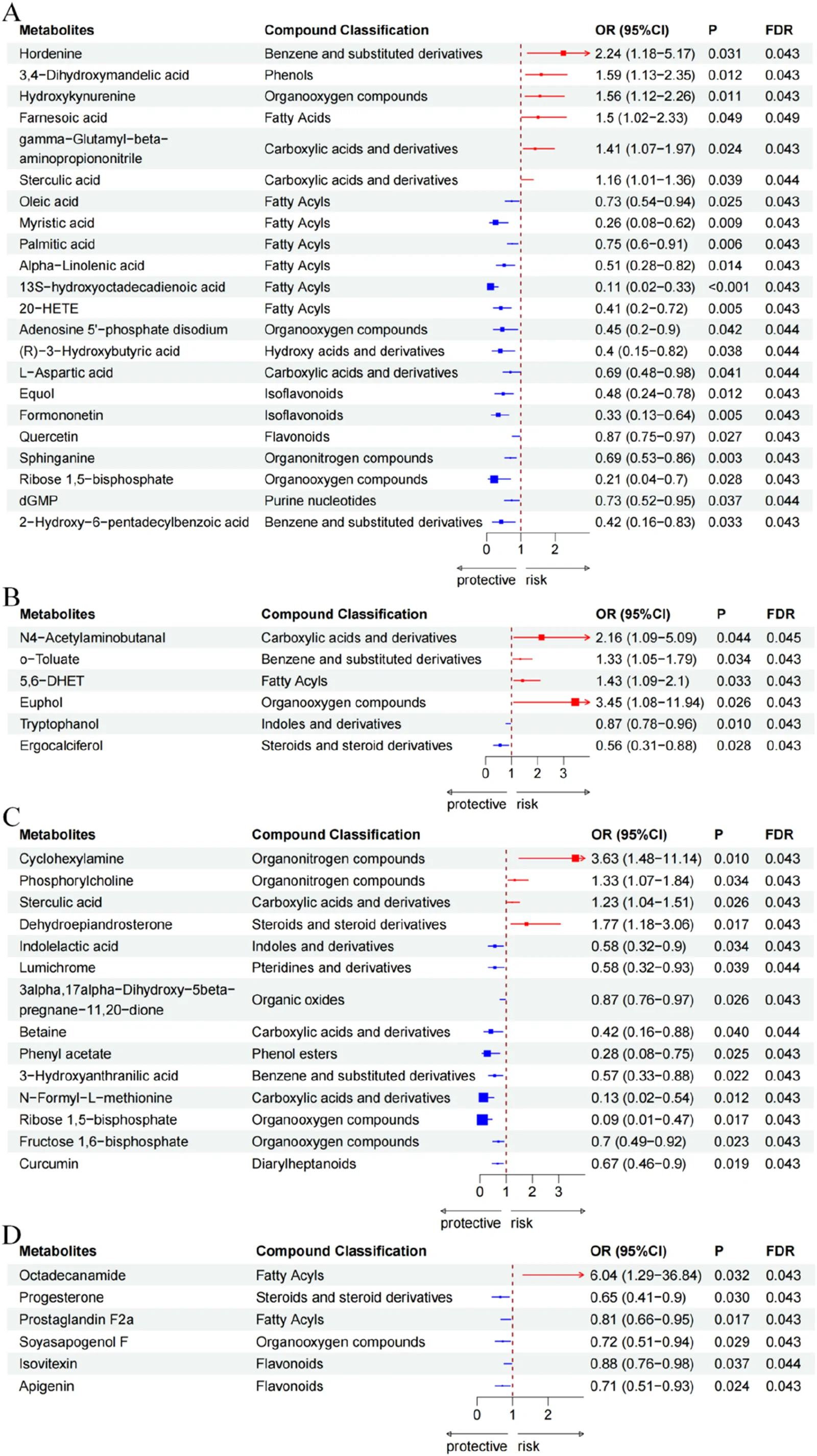
**Forest plots displaying the odds ratios (OR) and 95% Confidence Intervals (CI) for significant metabolites in:** (A) Maternal Plasma during Early-Mid Gestation (10–28 weeks); (B) Maternal Plasma in Late Gestation (38–40 weeks); (C) Umbilical Cord Blood; and (D) Neonatal Meconium. Metabolites are grouped by chemical class. An OR < 1 (left of the dashed line) indicates a protective effect, while an OR > 1 (right of the dashed line) indicates an increased risk. P-values were adjusted using the false discovery rate (FDR) method, and only associations with FDR <0.05 are reported. Metabolites identified as synthetic drugs were excluded

Fig. 3 identifies perinatal metabolic risk and protective factors associated with infantile AD. All metabolites discussed herein remained statistically significant after rigorous multiple testing correction (FDR <0.05). Notably, maternal plasma during early-mid gestation (Fig. 3A) exhibited the most profound metabolic alterations, revealing 22 significant metabolites. Among these, 16 metabolites exhibited protective associations with infantile AD. Specifically, these protective metabolites comprised 6 fatty acyls—highlighted by key PUFAs and anti-inflammatory lipid mediators (alpha-linolenic acid, 13S-hydroxyoctadecadienoic acid, and 20-HETE) alongside oleic, myristic, and palmitic acids—and 3 flavonoids/isoflavonoids (quercetin, equol, and formononetin). The remaining protective markers included 2 organooxygen compounds (ribose 1,5-bisphosphate and adenosine 5′-phosphate disodium), and 1 metabolite each from the following classes: purine nucleotides (dGMP), hydroxy acids and derivatives ((R)-3-hydroxybutyric acid), carboxylic acids and derivatives (l-aspartic acid), organonitrogen compounds (sphinganine), and benzene and substituted derivatives (2-hydroxy-6-pentadecylbenzoic acid). Conversely, 6 metabolites demonstrated risk associations: 2 carboxylic acids and derivatives (gamma-glutamyl-beta-aminopropiononitrile and sterculic acid), 1 phenol (3,4-dihydroxymandelic acid), 1 organooxygen compound (hydroxykynurenine), 1 fatty acid (farnesoic acid), and 1 benzene and substituted derivative (hordenine). To account for the broad sampling window during early-mid gestation (10–28 weeks), we conducted a sensitivity analysis by incorporating the exact gestational age at sampling as an additional covariate in the logistic regression (Supplementary Table S2). The associations for 21 of the 22 identified metabolites remained statistically significant (FDR <0.05), indicating that these core metabolic signatures are robust across this developmental window.

In contrast, maternal plasma in late gestation (Fig. 3B) contained only 6 statistically significant metabolites, indicating a diminished metabolic divergence prior to delivery. Among these, 2 metabolites exhibited protective associations: 1 steroid derivative (ergocalciferol) and 1 indole derivative (tryptophanol). The remaining 4 metabolites conferred elevated risk, comprising 1 fatty acyl (5,6-DHET), 1 organooxygen compound (euphol), 1 benzene and substituted derivative (*o*-toluate), and 1 carboxylic acid derivative (N4-acetylaminobutanal).

Umbilical cord blood (Fig. 3C) revealed 14 statistically significant metabolites. Among these, 10 metabolites demonstrated protective associations with infantile AD, including 2 organooxygen compounds (fructose 1,6-bisphosphate and ribose 1,5-bisphosphate), 2 carboxylic acids and derivatives (betaine and N-formyl-l-methionine), and 1 metabolite each from the following classes: benzene and substituted derivatives (3-hydroxyanthranilic acid), phenol esters (phenyl acetate), organic oxides (3alpha,17alpha-dihydroxy-5beta-pregnane-11,20-dione), diarylheptanoids (curcumin), indoles and derivatives (indolelactic acid), and pteridines and derivatives (lumichrome). Conversely, 4 metabolites demonstrated risk associations: 2 organonitrogen compounds (cyclohexylamine and phosphorylcholine), 1 steroid derivative (dehydroepiandrosterone), and 1 carboxylic acid derivative (sterculic acid).

Neonatal meconium (Fig. 3D) contained 6 statistically significant metabolites. Five of these demonstrated protective associations, comprising 2 flavonoids (isovitexin and apigenin), 1 steroid derivative (progesterone), 1 fatty acyl (prostaglandin F2α), and 1 organooxygen compound (soyasapogenol F). Conversely, 1 fatty acyl (octadecanamide) exhibited a risk effect.

Notably, 2 key compounds were concurrently identified in both early-mid gestation maternal plasma and umbilical cord blood: the antioxidant ribose 1,5-bisphosphate, which consistently exerted a protective effect, and sterculic acid, which was consistently identified as a risk factor. Furthermore, sterculic acid exhibited a significant positive maternal-fetal correlation specifically between early-mid gestation and cord blood (R = 0.44, P = 0.035). Collectively, these shared signatures and direct correlations indicate that the maternal metabolic milieu during early-mid gestation may exert a potential impact on fetal metabolites, highlighting this period as a critical window for defining infantile AD susceptibility.

### Pathway enrichment analysis

**Fig. 4:**
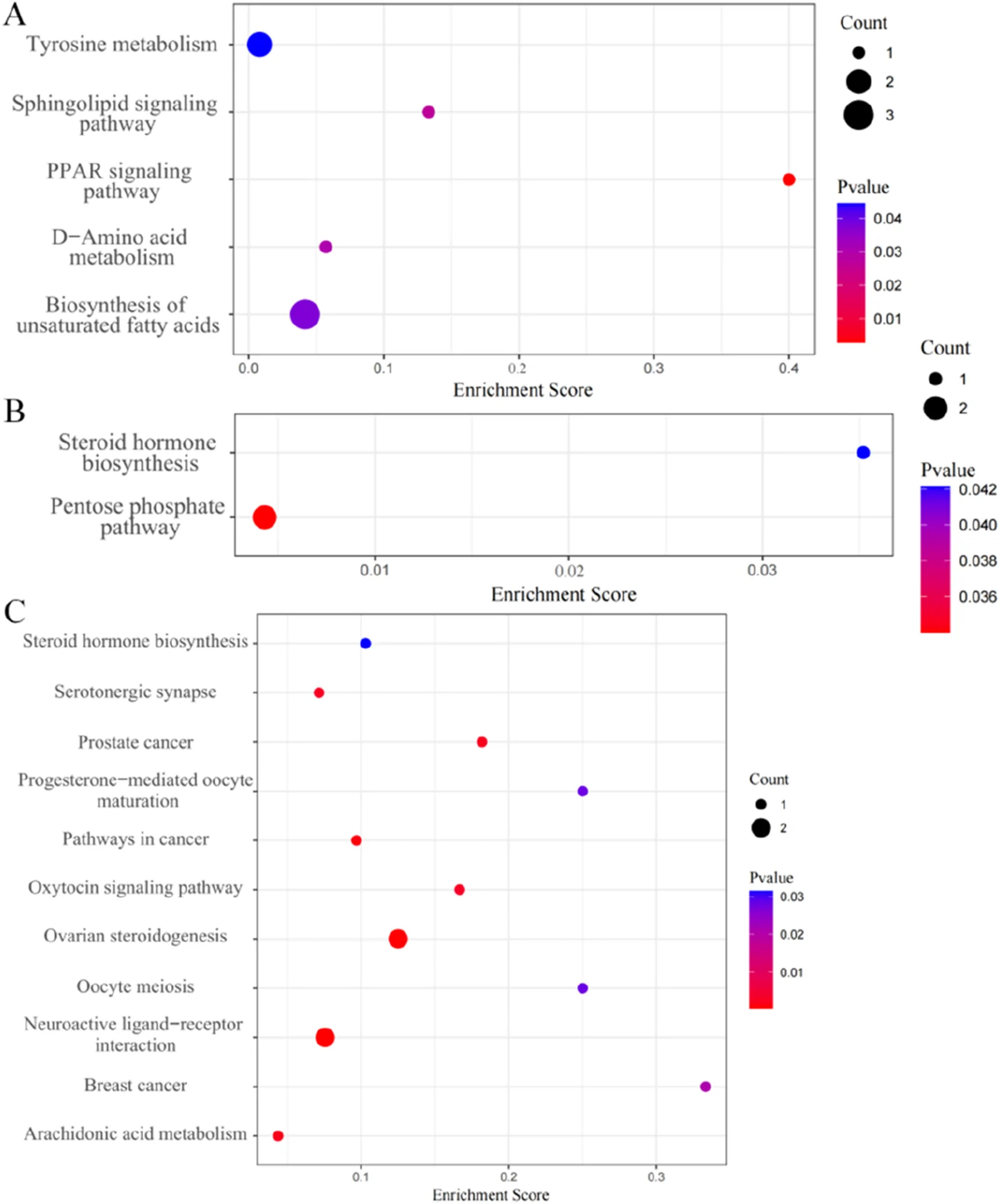
**KEGG pathway enrichment analysis of differential metabolites across perinatal matrices.** Bubble plots illustrate the key metabolic pathways altered in the AD group compared to the Healthy group. Enrichment analysis was performed using KEGG Compound mapping based on the significant metabolites identified via logistic regression in (A) Maternal Plasma during Early-Mid Gestation (10–28 weeks); (B) Umbilical Cord Blood; and (C) Neonatal Meconium. Node size represents the count of significant metabolites mapped to the pathway, and color intensity reflects the enrichment p-value

To elucidate the broader biological functions of the identified biomarkers, metabolic pathway enrichment analysis was performed. In maternal plasma during early-mid gestation (Fig. 4A), 5 metabolic pathways were significantly enriched. Consistent with our preceding observation highlighting the profound protective effects of fatty acyls and PUFAs, this early developmental window was heavily dominated by lipid metabolism and signaling cascades. Specifically, 3 of the 5 pathways were lipid-centric: the PPAR signaling pathway, the sphingolipid signaling pathway, and the biosynthesis of unsaturated fatty acids. The remaining 2 pathways were associated with amino acid metabolism (d-amino acid metabolism and tyrosine metabolism).

Umbilical cord blood (Fig. 4B) revealed 2 significantly enriched metabolic pathways: steroid hormone biosynthesis and the pentose phosphate pathway (PPP). Notably, the prominent enrichment of the PPP perfectly aligns with the consistent identification of the antioxidant ribose-1,5-bisphosphate as a key protective metabolite traversing the maternal-fetal circulation.

Neonatal meconium (Fig. 4C) revealed the most diverse pathway profile, with 11 pathways exhibiting statistical significance. While KEGG nomenclature categorized several of these pathways under “reproductive development” (eg, oocyte meiosis, progesterone-mediated oocyte maturation, ovarian steroidogenesis) and “cancer-related processes” (eg, breast cancer, prostate cancer, and pathways in cancer), biologically, these enrichments are primarily driven by the accumulation of fetal and maternal steroid hormones (such as progesterone) and lipid mediators in the fetal gut over the course of gestation. Furthermore, serotonergic synapse and neuroactive ligand-receptor interaction pathways were enriched, reflecting early neuroendocrine signaling. Finally, arachidonic acid metabolism was significantly enriched, underscoring the vital role of fetal gut inflammatory and lipid metabolic regulation in shaping early AD susceptibility.

To investigate the potential vertical transfer relationship, 5 metabolites were selected: ribose-1,5-bisphosphate, sterculic acid, 13S-hydroxyoctadecadienoic acid (13S-HODE), α-linolenic acid, and progesterone. Among these, only sterculic acid exhibited a significant positive correlation between maternal plasma during early-mid gestation and cord blood (R = 0.44, p = 0.035), suggesting its potential vertical transfer via the maternal-fetal circulation during this period. No significant correlations were observed among the remaining metabolites (P > 0.05).

### Random Forest modeling for the early prediction of infantile AD across distinct developmental windows

**Fig. 5:**
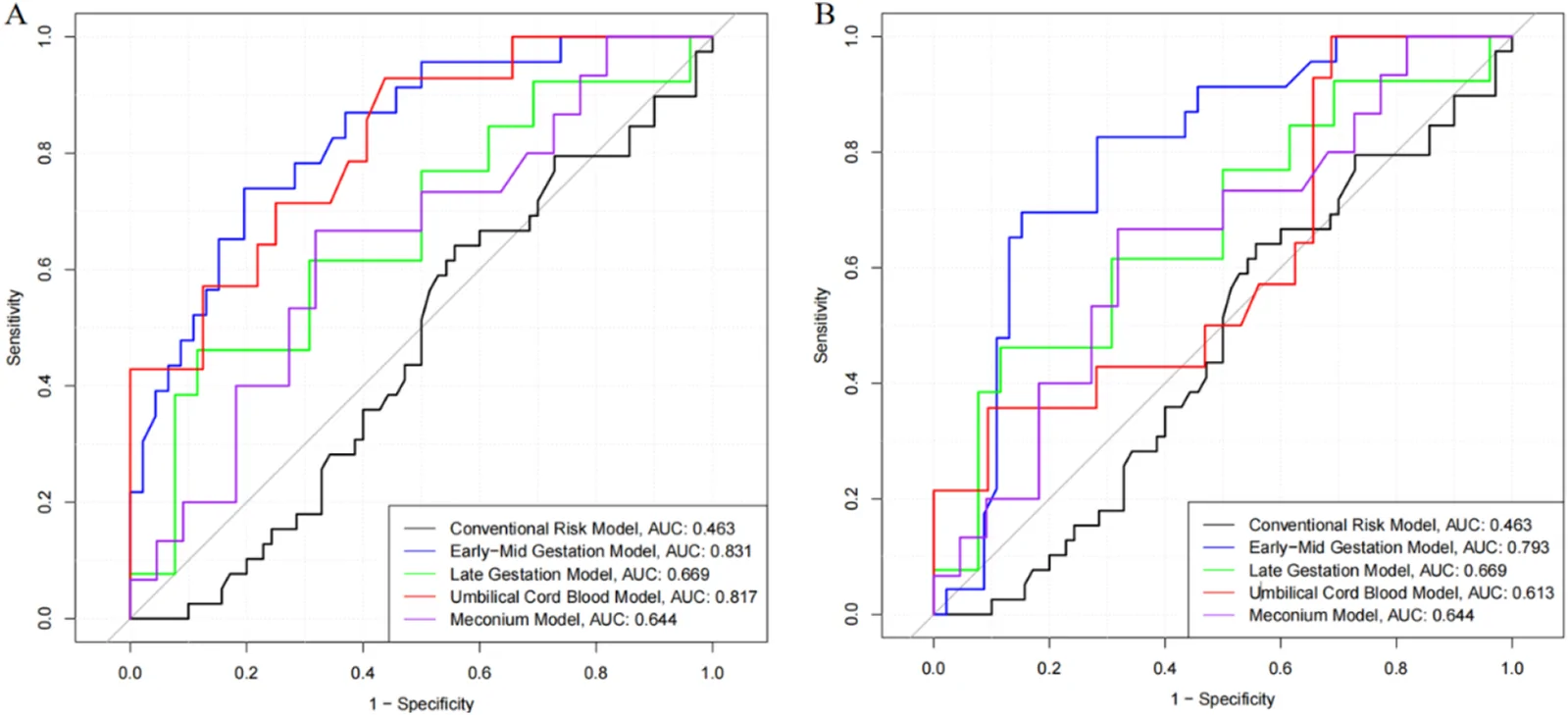
**Random Forest classification models for the early prediction of infantile Atopic Dermatitis (AD) across distinct perinatal time windows.** (A) Comprehensive Models: ROC curves comparing the predictive performance of models incorporating all significant differential metabolites identified in each respective biological matrix in Fig. 3, combined with conventional risk factors. (B) Parsimonious (Top 6) Models: A head-to-head comparison restricting the metabolic input to a maximum of the Top 6 most contributing metabolites (ranked by Variable Importance scores) plus conventional risk factors. This standardization eliminates statistical bias caused by unequal numbers of candidate biomarkers across different matrices. Model Definitions: Baseline Clinical Model (Black): Incorporating baseline demographic and familial factors (maternal age, pre-pregnancy BMI, and parental allergy). Early-Mid Gestation Model (Blue): Conventional factors combined with maternal plasma biomarkers (10–28 weeks). Late Gestation Model (Green): Conventional factors combined with maternal plasma biomarkers (38–40 weeks). Umbilical Cord Blood Model (Red): Conventional factors combined with cord blood biomarkers. Meconium Model (Purple): Conventional factors combined with meconium biomarkers. All models were evaluated using Leave-One-Out Cross-Validation (LOOCV).

Although the preceding analyses identified multiple stage-specific metabolic signatures associated with infantile AD, it remains unclear which developmental window provides the most robust statistical evidence for early disease prediction. To address this, we constructed Random Forest classification models utilizing Leave-One-Out Cross-Validation (LOOCV) to comprehensively evaluate and compare the predictive performance across the 4 biological matrices (Fig. 5).

We first established a Baseline Clinical Model incorporating baseline clinical characteristics (maternal age, pre-pregnancy BMI, and parental allergy). As expected, this model exhibited poor predictive performance (AUC = 0.463, 95%CI: 0.352–0.573), underscoring the inadequacy of relying solely on routine clinical histories for early AD forecasting.

Next, comprehensive predictive models were constructed by combining the conventional risk factors with all significant differential metabolites identified within each specific biological matrix. The integration of metabolic markers in Early-Mid Gestation Model and Umbilical Cord Blood Model significantly improved predictive accuracy over clinical characteristics alone (p < 0.001). Notably, the Early-Mid Gestation Model achieved an outstanding predictive accuracy (AUC = 0.831, 95%CI: 0.731–0.931), which vastly outperformed the Late Gestation Model (AUC = 0.669, 95%CI: 0.478–0.860). Within the neonatal matrices, the Umbilical Cord Blood Model (AUC = 0.817, 95%CI: 0.686–0.948) demonstrated superior predictive value compared to the Neonatal Meconium Model (AUC = 0.644, 95%CI: 0.459–0.829).

To rigorously validate these findings and eliminate potential statistical bias caused by the unequal number of candidate biomarkers across different matrices (eg, 22 metabolites in early-mid gestation vs. only 6 in late gestation), we further constructed parsimonious models. These models were strictly restricted to a maximum of 6 core metabolites, selected based on their Variable Importance scores (such as 13S-HODE, sphinganine, and sterculic acid; Supplementary Table 3). In this strictly feature-matched, head-to-head comparison, the predictive superiority of the Early-Mid Gestation Model remained highly robust (AUC = 0.793, 95%CI: 0.682–0.903). In contrast, the performance of the Cord Blood Model substantially declined (AUC = 0.613, 95%CI: 0.429–0.796) when restricted to only 6 features.

Overall, these results indicate that the maternal metabolic milieu during early-mid gestation harbors the strongest and most independent biological signals for AD susceptibility. The robust performance of its parsimonious model highlights early-mid gestation as the premier clinical window for developing streamlined, high-accuracy screening panels for infantile AD.

## Discussion

To our knowledge, this is the first longitudinal, multi-matrix metabolomic study to characterize perinatal metabolic signatures associated with infantile AD across distinct developmental windows. By systematically profiling maternal plasma (early-mid and late gestation), umbilical cord blood, and neonatal meconium, we identified a marked stage-specific metabolic divergence. The early-mid gestation period exhibited the most profound metabolic alterations—driven heavily by protective lipids and flavonoids—which subsequently diminished by late gestation. Crucially, our machine learning models established early-mid gestation as the optimal predictive window for infantile AD, demonstrating that the early maternal metabolic milieu plays a defining role in shaping fetal immune and barrier trajectories, providing objective biomarkers for early risk identification.

The longitudinal observation that profound metabolic divergence in early gestation diminishes by late gestation offers important biological insights. We hypothesize this trajectory is driven by 2 phenomena. First, the early gestational milieu strongly reflects baseline maternal dietary and inflammatory states during the highly sensitive window of initial fetal immune programming. However, by late gestation, the maternal system undergoes universal, profound physiological adaptations—such as generalized hyperlipidemia and metabolic shifts in preparation for parturition and lactation—which likely override and ‘mask’ the subtle early-stage metabolic differences associated with infant.^19^^,^^20^ Furthermore, the maturation of the placenta over time acts as a highly selective filter, actively regulating lipid and nutrient transfer,^21^ ultimately resulting in the distinct, refined metabolic profiles observed in cord blood.

A striking feature of the early maternal metabolic milieu was the significant enrichment of protective fatty acyls, particularly polyunsaturated fatty acids (PUFAs) such as alpha-linolenic acid (ALA) and 13S-HODE. These findings align with previous birth cohort studies associating prenatal maternal PUFA supplementation with a reduced risk of early-life.^22^^,^^23^ Biologically, PUFAs and their oxylipin derivatives act as potent anti-inflammatory mediators and are critical structural components of the developing epidermal lipid barrier.^24^^,^^25^ For instance, ALA serves as a vital precursor for immunomodulatory eicosanoids, while 13S-HODE has been shown to suppress pro-inflammatory cytokine production via PPAR-γ signaling pathways.^24^ Rather than exhaustively detailing single-molecule mechanisms, our findings robustly support the broader clinical premise that maintaining optimal maternal systemic PUFA levels during early pregnancy provides essential immunological and barrier protection against neonatal atopy.

Parallel to the lipid signatures, we identified a broad spectrum of protective flavonoids and isoflavonoids—including quercetin, formononetin, and equol—predominantly enriched in early-mid gestation maternal plasma. These plant-derived compounds primarily originate from maternal dietary intake of fruits, vegetables, and legumes. Flavonoids are well-documented for their capacity to mitigate oxidative stress, modulate immune chemokine expression, and preserve epithelial barrier homeostasis.^26^^,^^27^ Furthermore, compounds like equol, a microbial metabolite of dietary isoflavones, underscore the potential indirect influence of the maternal gut microbiome on the fetal immune environment.^28^^,^^29^ The prominent protective association of these dietary-derived metabolites represents a plausible avenue for future investigation of maternal nutritional interventions.

In addition to class-wide protective signatures, our study identified specific metabolites concurrently altered in both early-mid gestation maternal plasma and umbilical cord blood. Notably, the antioxidant ribose-1,5-bisphosphate emerged as a consistent protective marker. As a key intermediate in the pentose phosphate pathway (PPP), it plays an essential role in generating NADPH to combat oxidative stress, which is crucial for maintaining cellular integrity during early epidermal development.^30^^,^^31^ Conversely, sterculic acid was consistently identified as a risk factor, exhibiting a direct positive maternal-fetal correlation exclusively during early-mid gestation. Sterculic acid is known to inhibit stearoyl-CoA desaturase-1 (SCD1), an enzyme critical for lipid desaturation and homeostasis.^32^ While the exact mechanistic role of sterculic acid in AD pathogenesis requires future in vivo verification, its stage-specific correlation suggests that fetal exposure to disrupted lipid homeostasis during this critical early window may compromise barrier maturation.

A major strength of our study is the translation of these multi-matrix metabolic signatures into actionable predictive models. Current risk assessments for infantile AD typically rely on baseline clinical factors (eg, family history of atopy) which, as demonstrated in our Baseline Clinical Model (AUC = 0.463, 95%CI: 0.352–0.573), provide inadequate predictive accuracy. Recent advances in machine learning have utilized post-natal biomarkers—such as infant gut microbiome profiling^33^ or neonatal skin tape-strip chemokines collected at 2 months of age^34^—to predict AD development, achieving AUCs ranging from 0.73 to 0.91. However, our prenatal Early-Mid Gestation Model achieved highly competitive predictive accuracy (AUC = 0.831, 95%CI: 0.731–0.931) months before the infant's birth. Furthermore, this predictive superiority remained remarkably robust (AUC = 0.793, 95%CI: 0.682–0.903) even in a parsimonious model restricted to only 6 core metabolites. This finding is of substantial clinical significance, demonstrating that a streamlined, non-invasive maternal blood test during early pregnancy can accurately forecast infant AD, providing a crucial temporal window for early preventative strategies.

While this study benefits from a longitudinal design and robust machine learning validation, several limitations warrant consideration. First, the single-center nature of the cohort with 109 mother-infant pairs may limit the broad generalizability of the findings across diverse geographic and ethnic populations. Second, untargeted metabolomics provides relative quantification; future studies utilizing targeted LC-MS are required to establish absolute concentration thresholds for clinical diagnostic use. Third, a major limitation is the lack of an independent, external validation cohort. Although we employed rigorous internal cross-validation (LOOCV) to mitigate overfitting, the absence of external validation means our predictive models require confirmation in larger, multi-center populations before clinical translation. Finally, given the observational nature of the survey, the biological mechanisms linking specific metabolites to AD pathogenesis remain associative. Subsequent in vitro and in vivo models are needed to definitively verify their functional immunomodulatory roles.

## Conclusion

This longitudinal, multi-matrix metabolomic study demonstrates that the maternal metabolic milieu during early-mid gestation is strongly associated with infantile AD susceptibility. While protective signatures—driven predominantly by PUFAs and dietary flavonoids—and cross-matrix markers like ribose-1,5-bisphosphate and sterculic acid were identified, the most critical finding lies in their temporal predictive value. Crucially, our machine learning models established early-mid gestation as the optimal clinical window for early AD prediction. A parsimonious predictive model utilizing only 6 core maternal metabolites from this early stage achieved robust accuracy (AUC = 0.793), vastly outperforming conventional clinical risk factors and late-gestation profiles. These findings shift the paradigm of infantile AD risk assessment from post-natal observation toward prenatal prediction. However, the predictive associations between the early-mid gestation metabolome and AD remain observational; they require confirmation in future mechanistic and interventional trials before they can inform targeted nutritional strategies during pregnancy.

## Authors’ information

Xi Fu authors’ consent for publication: obtained; author contributions: investigation, analysis and interpretation, visualization, writing, and editing.

Zekai Liang authors’ consent for publication: obtained; author contributions: investigation, analysis and interpretation, visualization, writing, and editing.

Chuchu Luo authors’ consent for publication: obtained; author contributions: investigation, analysis and interpretation, writing, and editing.

Yuwei Tang authors’ consent for publication: obtained; author contributions: investigation, analysis, interpretation, and editing.

Xin Zhang authors’ consent for publication: obtained; author contributions: investigation, interpretation, and project administration.

Guo-Qing Fang authors’ consent for publication: obtained; author contributions: investigation, interpretation, and project administration.

Da-Yong Zha authors’ consent for publication: obtained; author contributions: investigation, interpretation, and project administration.

Yu Sun authors’ consent for publication: obtained; author contributions: conceptualization, methodology, interpretation, supervision, writing, review, and project administration.

All authors read and approved the final version of the article.

## Ethics approval

The study protocol was approved by the Ethics Committee of the first affiliated hospital of Guangdong Pharmaceutical University (Medical Ethics [2023] No.40).

## Disclosure of the use of generative AI and AI-assisted technologies

During the preparation of this work, the authors used Google Gemini in order to improve readability and optimize the English language. After using this tool, the authors carefully reviewed and edited the content as needed and take full responsibility for the ultimate content of the publication.

## Funding

This study was funded by the 10.13039/501100001809National Natural Science Foundation of China (42377106, 42307541), the 10.13039/501100003453Natural Science Foundation of Guangdong Province (2024A1515012476).

## Declaration of competing interest

The authors declare that the research was conducted in the absence of any commercial or financial relationships that could be construed as a potential conflict of interest.
